# The Czar's Illness and Its Lessons

**Published:** 1894-11-17

**Authors:** 


### The Czar's Illness and Its Lessons.

The report now published of the post-mortem examination of the late Czar of Russia tells us that the cause of death was chronic interstitial nephritis, accompanied by the usual hypertrophic cardiac affection. The picture thus presented of a man in the highest position dying of a disease which must have existed for years, and which yet seems only to have been discovered a few months before the end, draws attention to a subject which medical men will do well to note with care, viz., the importance of including an examination of the urine in the ordinary clinical routine. Perhaps it may be difficult or impossible to examine the urine in every case. A man pops in at a surgery on his way to the station, a lady is seen in a drawing-room, and all kinds of difficulties are apt to be raised, but, nevertheless, it remains true that no examination can be considered complete which omits the urine. It may be worth while then to insist on a few of the symptoms which demand with more than ordinary urgency such an examination. Of course all complaints of abnormalities in the secretion itself, or in the manner of its discharge, will receive proper attention. That ought to go without saying. Then all cases in which the slightest dropsy or puffiness of the ankles is discovered will go into the same category, and all cases in which complaints are made of either violence or irregularity of cardiac action. The relation of steadily increasing hypertrophy and tense arteries with chronic granular kidneys is recognised well enough, and no one would fail to inquire about the kidneys if he had before him a man with hard arteries and a heart showing signs of hypertrophy. It is not so generally recognised, however, that a heart may hypertrophy very little before it begins to fail, and that with only slight preliminaries the final act in the drama may be introduced as a mere intermittency or irregularity, an apparently functional disorder of a heart showing otherwise but few signs of disease. Moreover, a heart partially crippled by some youthful rheumatism but restored to usefulness by good compensatory hypertrophy may ultimately fail, not from lessening power in its muscle, but from the increase in its work due to a latent kidney affection, and thus again we see that heart symptoms which may appear self-explanatory may really require investigation of the urine for their complete elucidation. Recurring headaches, nausea and vomiting, transient attacks of giddiness, convulsive attacks, however much they may seem to be caused by drink or indigestion, all suggest albuminuria as a possible cause. While shortness of breath and loss of flesh, however occurring, should lead to careful examination of the urine, especially if complicated with apparently causeless attacks of nocturnal asthma or dyspnœa. The more carefully one studies the wide ramifications of the symptoms produced by imperfect action of the kidneys, the more one sees the necessity of being constantly awake to the possibility of even the simplest cases being instances of latent Bright's disease.

